# MIMO 5G Smartphone Antenna with Tri-Band and Decoupled Elements

**DOI:** 10.3390/s23115186

**Published:** 2023-05-30

**Authors:** Jianqiang Hou, Yuxuan Peng, Jiajun Huang, Zhefei Wang, Tayeb A. Denidni

**Affiliations:** 1 The School of Electronic Engineering, Xidian University, Xi’an 710071, China; houjq@163.com; 2 The School of Electronic and Information Engineering, Nanjing University of Information Science and Technology, Nanjing 210044, China; 202212490464@nuist.edu.cn (Y.P.); 202212490224@nuist.edu.cn (J.H.); 3 Institut National de la Recherche Scientifique, Université du Quebec, Montreal, QC H5A1K6, Canada; denidni@emt.inrs.ca

**Keywords:** miniaturization, decoupled antennas, tri-band, 4G/5G application

## Abstract

In this article, a miniaturized antenna is proposed for 4G/5G multiple input, multiple output (MIMO) applications for smartphones. The proposed antenna is composed of an inverted L-shaped antenna with decoupled elements to cover 4G (2000–2600 MHz), and a planar inverted-F antenna (PIFA) with a J-slot to cover 5G (3400–3600 MHz and 4800–5000 MHz). Furthermore, to achieve the purposes of miniaturization and decoupling, the structure adopts a feeding stub, shorting stub, and outstanding floor, additionally adding the slot to the PIFA, to generate additional frequency bands. Due to the advantages such as multiband operation, MIMO configuration for 5G communications, high isolation, and a compact structure, the proposed antenna design is attractive for 4G/5G smartphones. The antenna array is printed on an FR4 dielectric board, measuring 140 × 70 × 0.8 mm^3^, with the 4G antenna located on a top 15 mm-long headroom.

## 1. Introduction

The latest smartphones generally support 5G networks, which can provide mobile phone users with voice calls and mobile data services with less delay and greater bandwidth to meet users’ daily communication needs. Thus, 5G technology has been widely used in the smartphone antenna industry [1,2,3]. However, the current 5G network coverage is low, and due to the relatively large attenuation of high-frequency signals, smartphones still need to fall back to 2G/3G/4G networks for communication in areas with poor or no coverage of 5G networks. The difficulty in the MIMO antenna design of mobile devices is that the space available for antennas is very limited, and signals are prone to interference, especially when the antenna is added to a 4G antenna [4,5]. This causes great design challenges for the internal antennas in modern smartphones.

Recently, several MIMO antennas for mobile devices have been proposed. In [6,7], an 8-element 5G MIMO antenna is mentioned, and the place for a 4G antenna is reserved. In [8], a novel PIFA antenna with a slot is proposed, in order to add a new resonant frequency. Moreover, others used a hybrid of a Franklin strip monopole antenna and a rectangular patch antenna [9], the design of a dual feature of the rectangular open-ended ground slot is proposed in [10], and the structure of an outstanding ground to decouple is used in [11,12,13]. Four conductive, innovatively designed antenna elements are diagonally placed on the flexible polyamide substrate, which could keep operating when the antenna is bent in [14]. The antenna consists of an Annular Ring structure with a CPW-Fed operating in both the sub 6 GHz 5G as well as WLAN band in [15]. The Wideband Low-Profile antenna is designed to match Ultrathin 5G Smartphones in [16]. A novel wideband self-decoupled loop-coupled antenna element is proposed for the 5G MIMO smartphones in [17]. Ref. [18] inserts a rectangular slot under each microstrip feed line to obtain a wideband. Yet, all of the articles do not take into account the isolation, coupling, and miniaturization of 4G antennas and 5G antennas. This paper combined the structure of 4G and 5G, and considered the coupling and isolation between antennas.

In this paper, a tri-band 8-element MIMO antenna is proposed. Besides the ability to cover the 5G bands n78 and n79, it can also yield the 4G band (2000–2700 MHz). In addition, it can exhibit desirable impedance matching and isolation (>10 dB) across the three operating bands, and the envelope correlation coefficient (ECC) of the 8-element antennas is also calculated [19]. HFSS is used to simulate the performance of parameters. The numerical method of the characteristic modes analysis is used in the antenna design.

## 2. Proposed 8-Element MIMO Antenna Structure

**Figure 1:**
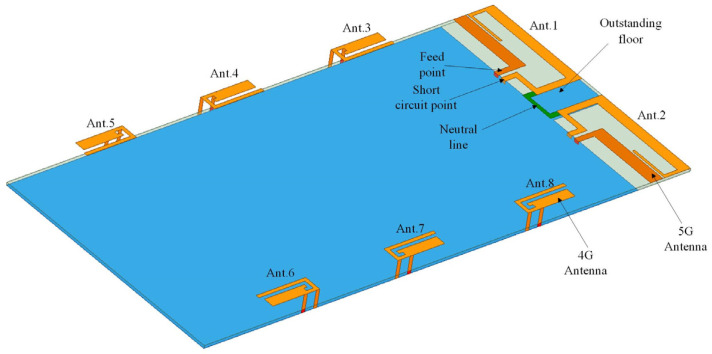
Geometry of the 8-element antenna.

Figure 1 shows the detailed geometry and dimensions of the proposed MIMO antenna. The antenna array is printed on an FR4 dielectric board with a size of 140 × 70 × 0.8 mm^3^, and the 4G antenna is located on a 15 mm long clearance area on the top, which prominently reduces the coupling between units. The 5G antennas are evenly arranged on both sides of the back panel of the mobile phone. The unit is a planar inverted F antenna, so no additional headroom is required. A new current path is constructed through the loading gap, so that the antenna can work in two frequency bands.

In this paper, by optimizing parameters such as feeder width, protruding ground size, antenna spacing, and antenna width, length, and height, the parameters such as return loss, isolation, and the radiation pattern of the antenna in the working frequency band can be optimized.

### 2.1. 4G Section

**Figure 2:**
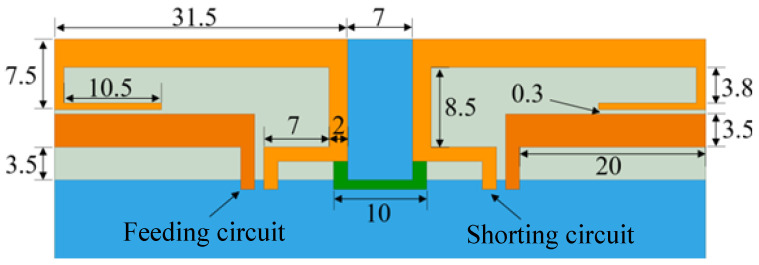
4G antenna elements.

The 4G antenna consists of four parts: the feeding stub, short-circuit stub, protruding ground, and neutral line. The clearance area used is 15 × 70 mm^2^. The specific size is shown in Figure 2.

Each antenna unit is composed of a feed stub and a short-circuit stub. The short-circuit stub generates a new resonance frequency point outside the working frequency band of the feed stub, which expands the antenna’s working bandwidth. By optimizing the length of the short-circuit end stub, it can be adjusted. The width of the feeding line and the shorting line are both 1.5 mm, and the characteristic impedance of the microstrip line is 50 Ω at this time.

The protruding ground structure and the neutral line loaded between the antenna elements can improve the isolation between the antenna elements. Among them, the width of the protruding ground is 10 mm, and the distance between two short-circuit branches is 7 mm. The overlapping width between the short-circuited stub and the protruding floor is 1.5 mm, and the capacitive coupling generated can reduce the resonant frequency of the antenna and achieve the purpose of miniaturization.

**Figure 3:**
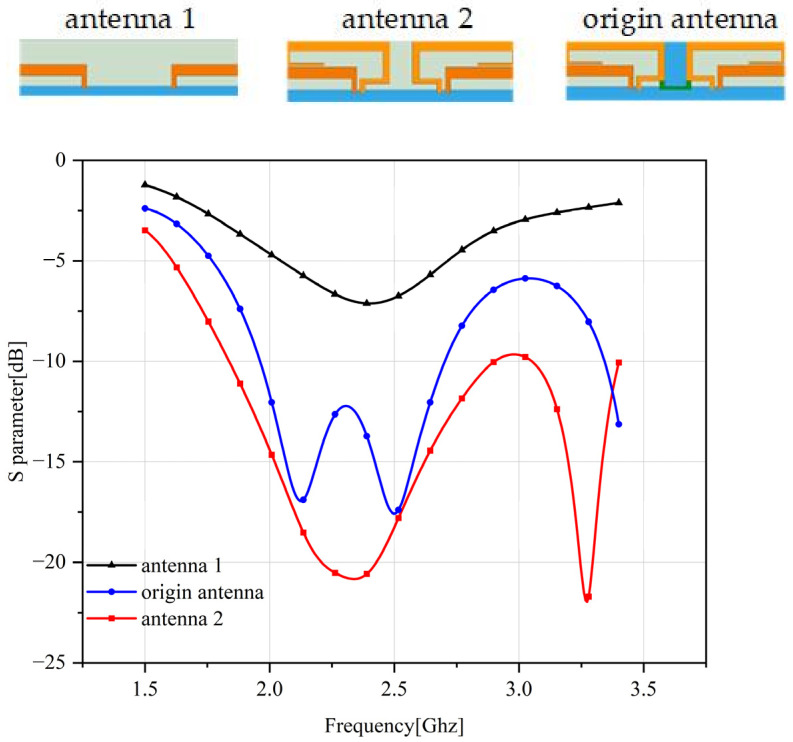
4G Antenna Structure and Return Loss.

When the antenna unit has no short-circuit stubs and only monopole radiation, refer to antenna 1 in Figure 3; the working frequency of the antenna is 2500 MHz. Due to poor matching currently, the antenna return loss is only 7 dB.

By adding a short stub, such as reference antenna 2 in Figure 3, the matching of the antenna can be improved and a new resonant frequency point at 3200 MHz can be generated. Yet, because the distance between the two short-circuit stubs is too small, the coupling between the antenna elements is relatively large. At the short-circuit stub resonant frequency, the isolation is only 8.5 dB.

Adding a neutral line and a protruding ground structure between the antenna units can improve the isolation between the antenna units: the coupling between the antenna and the protruding ground can reduce the coupling between the antenna units, and the conduction current on the neutral line can be coupled with the radiation The current is neutralized to achieve a decoupling effect.

Due to the 1.5 mm wide overlap between the short stub and the salient ground, the resulting capacitive coupling shifts the resonant frequency of the short stub to a lower frequency, from 3200 MHz without a salient ground to 2100 MHz. The operating frequency of the monopole is 2500 MHz, and the added short-circuit stub can expand the bandwidth of the antenna.

**Figure 4:**
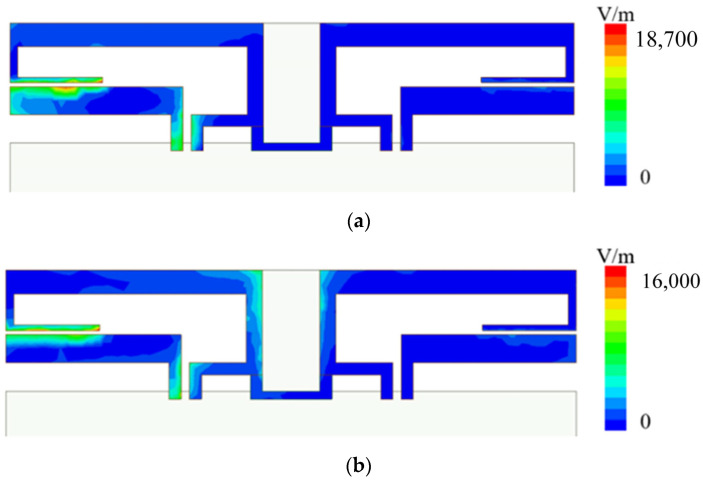
(**a**) Electric field distribution at 2100 MHz. (**b**) Electric field distribution at 2500 MHz.

The electric field distribution of the antenna when it is working is shown in Figure 4.

When the frequency is 2100 MHz, the feed stub excites the short-circuited stub through coupling, and the short-circuited stub radiates outward. Due to the short distance of the short-circuited stub, the coupling degree of the antenna reaches the maximum at the resonance frequency of the short-circuited stub at 2100 MHz. However, due to the addition of additional decoupling stubs, the maximum coupling degree is only 14.7 dB. When the operating frequency is 2500 MHz, it mainly radiates outwards from the feeding stubs, as shown in Figure 4. Due to the long distance between the two feeding stubs and the existence of decoupling stubs, the isolation between antenna elements reaches 17.5 dB.

**Figure 5:**
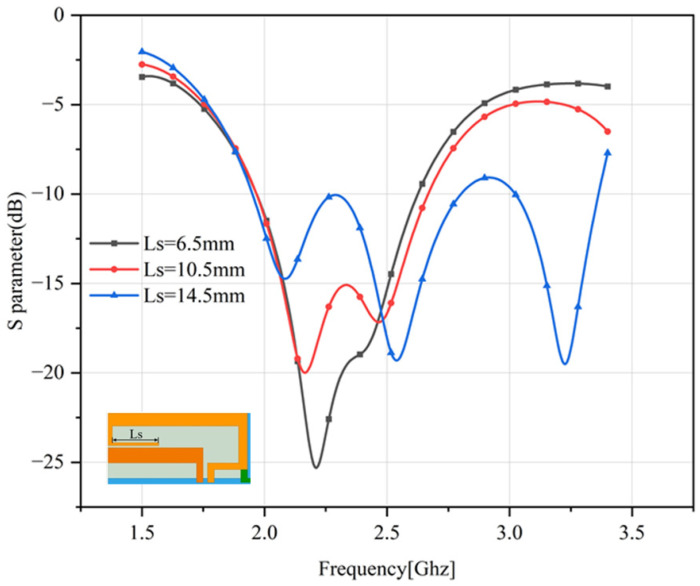
The influence of terminal length on the resonant frequency.

By adjusting the length of the terminal branch of the short-circuited stub, the resonant frequency of the short-circuited stub can be tuned, as shown in Figure 5. When the antenna terminal stub length Ls increases from 6.5 mm to 14.5 mm, the antenna resonant frequency shifts from 2210 MHz to 2080 MHz. Through optimization, the value of Ls is taken as 10.5 mm, so that the resonance frequency of the short-circuited stub is 2100 MHz.

By adding short-circuit stubs and decoupling structures to the antenna, the working bandwidth, return loss, and coupling degree of the antenna unit are optimized. The scattering parameters of the antenna are shown in Figure 5. The 10 dB working frequency band of the antenna is 2000–2700 MHz, and the isolation in the working frequency band is greater than 13 dB.

### 2.2. 5G Section

The MIMO antenna proposed in this chapter contains a total of six 5G antenna elements. Since the 4G antenna uses a clearance area with a length of 15 mm, the area used by the 5G antenna is 125 × 70 mm^2^. There are three symmetrical 5G antenna units on each side of the metal backplane with a spacing of 23 mm, and the distance between the antenna units on both sides is 19 mm from the edge of the backplane.

**Figure 6:**
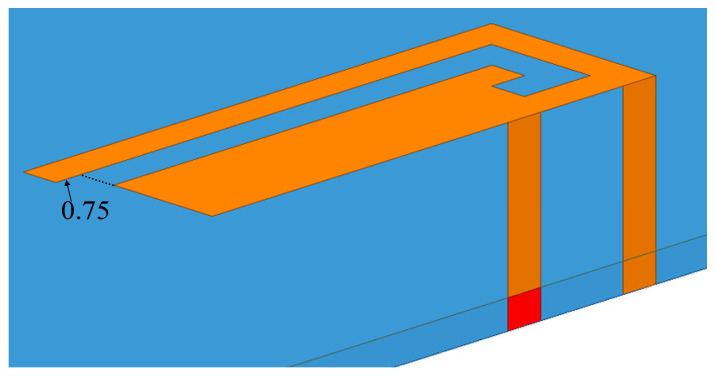
5G Antenna structure.

The antenna structure is a planar inverted-F antenna loaded with slots, as shown in Figure 6. The PIFA antenna has the advantages of low profile, easy matching, etc., and does not require additional headroom. The bottom of the antenna can be a complete metal floor, which is suitable for a mobile phone antenna with a high screen ratio.

The height of the PIFA antenna in this chapter is 5 mm, the distance between the feed point and the short-circuit point is 2.5 mm, and the plane size is 14.2 × 5 mm^2^. A J-shaped slot with a length of 13.5 mm and a width of 1 mm is loaded on the plane of the PIFA antenna. The gap creates a new current path that allows the antenna to operate at two frequencies.

From the feed point to the open end along the J-shaped slot, a longer current loop is formed; from the feed point directly to the open end of the PIFA antenna, a shorter current loop is formed. Tuning the lengths of the two current loops separately can adjust the resonant frequency points of the high and low frequencies. For the antenna in this chapter, the stub is extended by 0.75 mm at the end of the J-shaped slot to optimize its resonant frequency, as shown in Figure 6.

**Figure 7:**
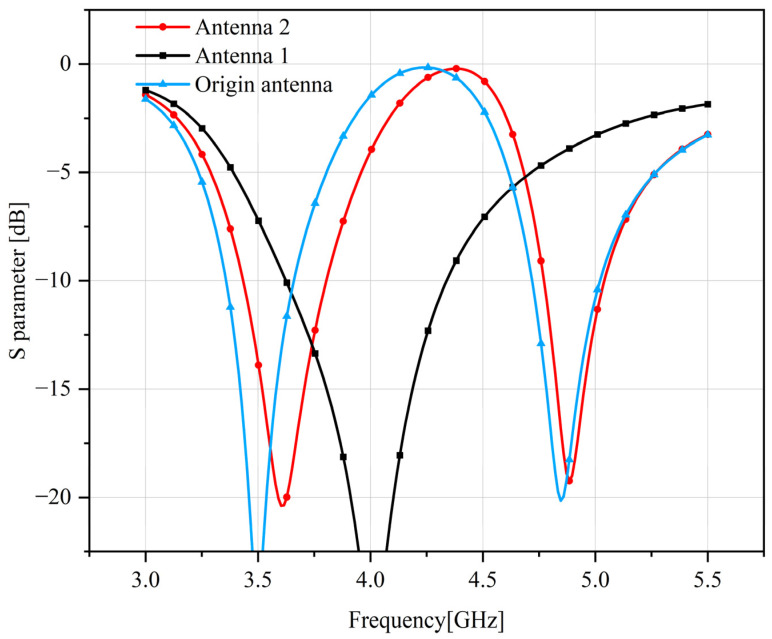
5G Antenna structure and return loss.

The prototype of the antenna is a common PIFA antenna, the height of the antenna is 5 mm, the plane size of the antenna is 15 × 3 mm^2^, and the distance between the feed point and the short-circuit point is 2.5 mm; a resonant frequency point can be generated at 4000 MHz, and the working bandwidth of the antenna is 700 MHz, which is well matched, and it is shown as Antenna 1 in Figure 7. By loading a J-shaped slot on the plane of the above-mentioned PIFA antenna, a new path can be constructed for the current, so that the antenna generates a new resonance frequency point at 5000 MHz. However, because the gap destroys the original current path and increases a new current path, the original resonant frequency of the antenna is shifted to a lower frequency of 3600 MHz. That is, a dual-band PIFA antenna working at 3600 MHz and 5000 MHz is obtained at this time, as shown in Figure 7, referencing antenna 2. From the feed point to the open end along the J-shaped gap, a longer current loop is formed, and the loop works in the 3600 MHz frequency band. From the feed point directly to the open end of the PIFA antenna, a shorter loop is formed, and the loop works in the 5000 MHz frequency band.

**Figure 8:**
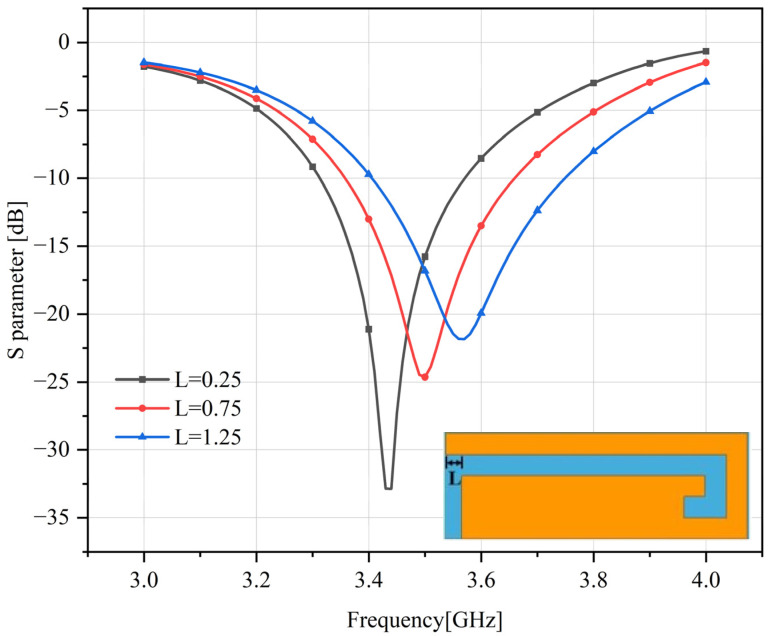
Effect of end length on antenna resonant frequency.

Since the antenna is expected to operate at 3500 MHz, the resonant frequency of the longer branch of the antenna can be tuned by optimizing the length *L* of the open stub end in reference antenna 2. As *L* increases, the resonant frequency of the longer branch of the antenna moves to lower frequencies, as shown in Figure 8 and the origin antenna of Figure 7. When *L* = 0.75 mm, the resonance frequency of the antenna shifts from 3600 MHz to 3500 MHz.

**Figure 9:**
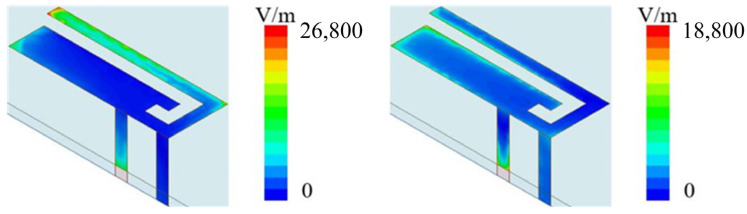
Electric Field Distribution of 5G Antenna.

After optimization, the two operating frequencies of the PIFA antenna are 3400–3600 MHz and 4800–5000 MHz, which can cover the frequency bands of n79 and n78. The electric field distribution of the antenna is shown in Figure 9. When the antenna operates at 3500 MHz, it radiates outwards from the long current loop; when the antenna operates at 5000 MHz, the antenna radiates outwards from the short current loop.

**Figure 10:**
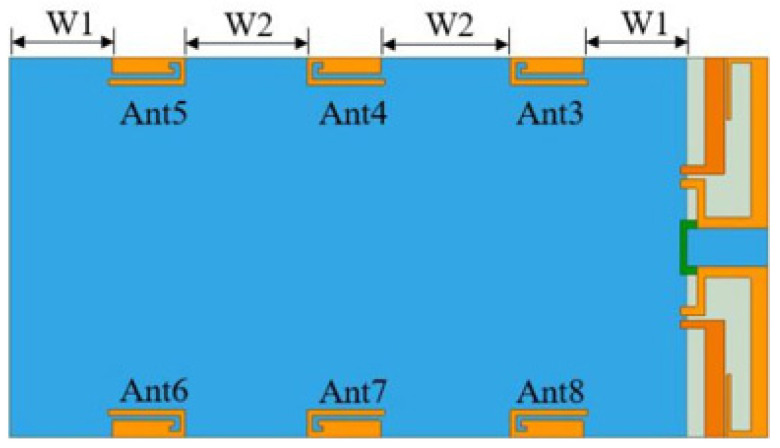
Distance of antenna to floor.

The PIFA antenna does not require a clear space but radiates equivalently through the reflection image of the floor, so a relatively complete floor is required under the PIFA antenna. Therefore, the distance between the antenna unit and the edge of the floor will affect the radiation performance of the antenna. As shown in Figure 10, the greater the distance *W*_1_ between the antenna unit and the edge of the floor, the better the radiation effect. However, the increase of *W*_1_ will make the antenna spacing *W*_2_ too small, which will increase the coupling between adjacent antenna elements. By optimizing the values of *W*_1_ and *W*_2_, the radiation performance of the PIFA antenna can be optimized and the isolation between antenna elements can reach more than 10 dB.

When *W*_1_ increases, that is, when antenna 3 and antenna 5 are far away from the edge of the floor, the impedance matching and radiation performances of the antenna are optimized. However, due to the relative reduction of *W*_2_, that is, the distance between the antenna elements is reduced, the coupling between the antenna elements is too large, and the isolation is less than 10 dB. When *W*_1_ is reduced, the matching of the antenna gradually deteriorates but the isolation between elements is optimized. After a comparative analysis, the value of *W*_1_ is 19 mm and *W*_2_ is 23 mm. Currently, the matching of each antenna is good, and the isolation between units is greater than 10 dB.

**Figure 11:**
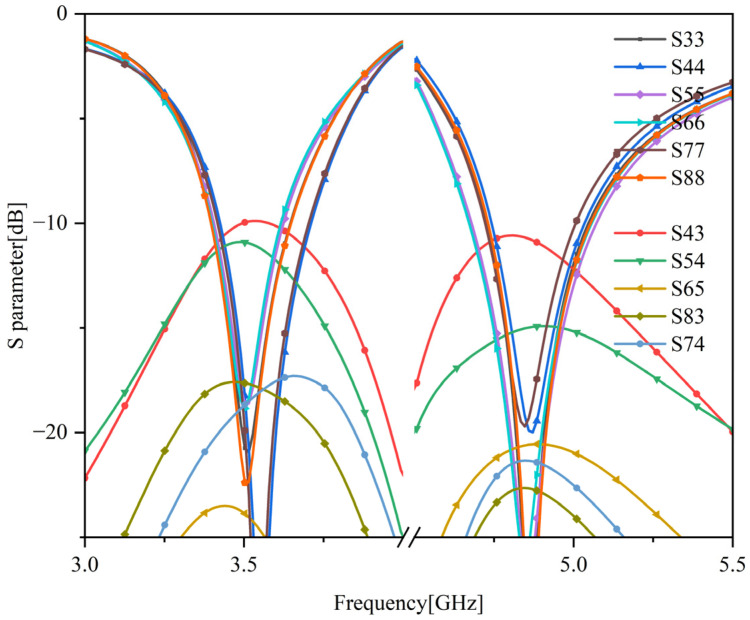
Scattering parameters of 5G antennas.

Based on the above analysis, the basic structure and distribution position of the 5G antenna elements have been determined. After simulation, the scattering parameters of the six-element MIMO antenna are shown in Figure 11. According to the simulation results of scattering parameters, the mobile phone MIMO antenna in this section can work in the two frequency bands of 3400–3600 MHz and 4800–5000 MHz, and the return loss of the antenna unit within the working bandwidth is greater than 10 dB. The isolation between adjacent antennas on the same side is greater than 10 dB, and the isolation between symmetrically adjacent antenna elements on both sides is greater than 17 dB.

## 3. Results

**Figure 12:**
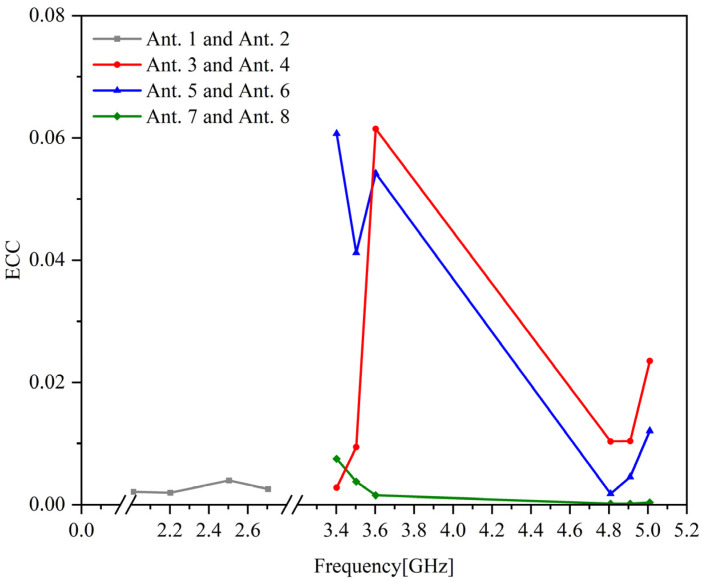
Envelope correlation coefficient of MIMO antennas.

The envelope correlation coefficient is one of the important parameters of the MIMO antenna, which reflects the diverse characteristics of MIMO. The ECC value of the low-frequency band of the antenna is required to be less than 0.5, and the ECC value of the high-frequency band is less than 0.4. The MIMO toolkit in the HFSS software can calculate the envelope correlation coefficient of the antenna according to the scattering parameters obtained by simulation. It can be seen in Figure 12 that the ECC values between the antenna units are all less than 0.1, the ECC values of the two 4G antenna units in the working frequency band are below 0.05, and the ECC values between the 5G antenna units are below 0.1. It shows that the MIMO antenna proposed in this chapter has better diversity characteristics, smaller diversity gain, and smaller signal distortion due to multipath effects. Since the ECC value obtained by simulation is an ideal result, the ECC value of the antenna in the actual working process will be slightly larger.

The cell phone antenna needs to be close to the human hand or head when it works, and the human body as a conductor has a greater influence on the radiation performance of the antenna.

**Figure 13:**
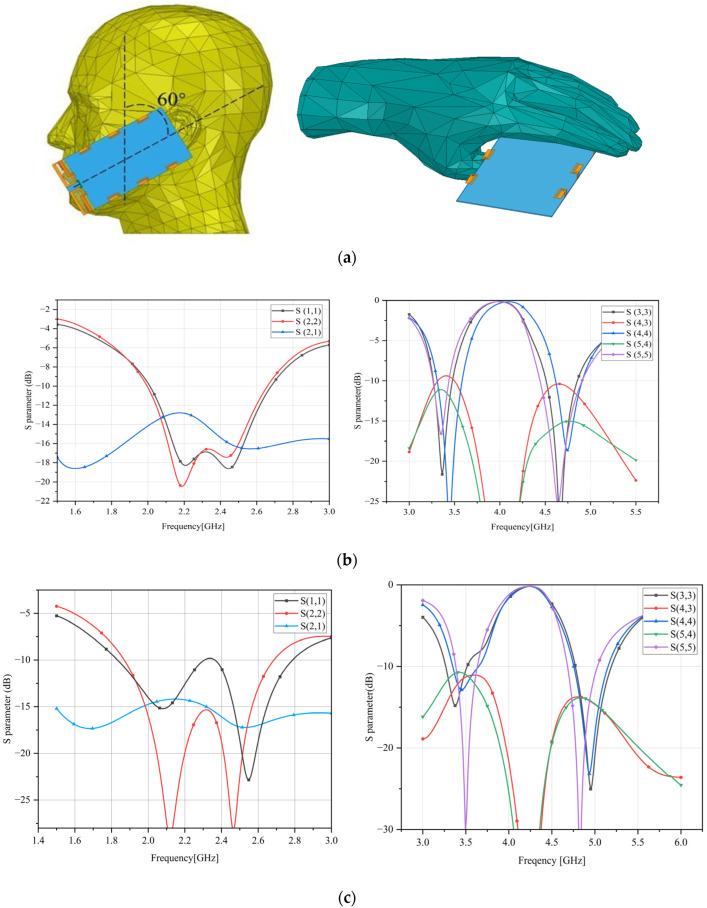
(**a**) Head or hand model. (**b**) The effect of the head on the antenna. (**c**) The effect of the hand on the antenna.

Through the hand or head model simulation, we can illustrate the influence of the human body on the antenna radiation performance, whether the antenna can work normally when the phone is held or talking. As in Figure 13 when the phone is tilted 60°, 1 mm away from the human head, and when the phone is held by the hand on the lower part, the impact of the head or hand on the antenna scattering parameters in the talking state is simulated.

From the simulation results, the coupling between 4G antenna units is enhanced due to the reflection of the head or hand, the isolation is reduced to 12 dB, and the return loss of 4G antenna is increased. This is not because the matching of the antenna is optimized, but because the head absorbs part of the electromagnetic energy radiated outward by the antenna. The resonant frequency of the 5G antenna is slightly shifted, and the analysis shows that this is because the head is equivalent to a conductor, the frequency deviation of the PIFA antenna caused by a change in the shape of the floor below it. The isolation between the 5G antenna units is also reduced, also due to the reflection from the head or hand.

**Figure 14:**
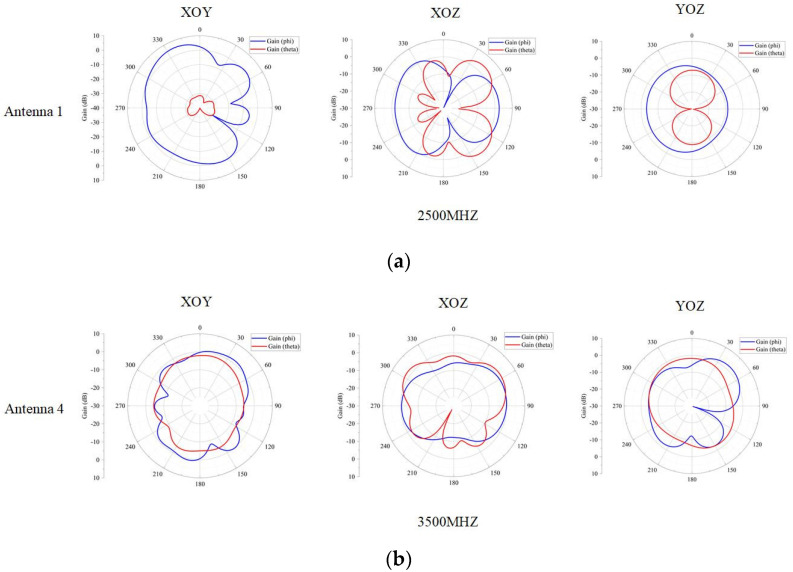
(**a**) Radiation pattern of the antenna 1. (**b**) Radiation pattern of the antenna 4.

**Figure 15:**
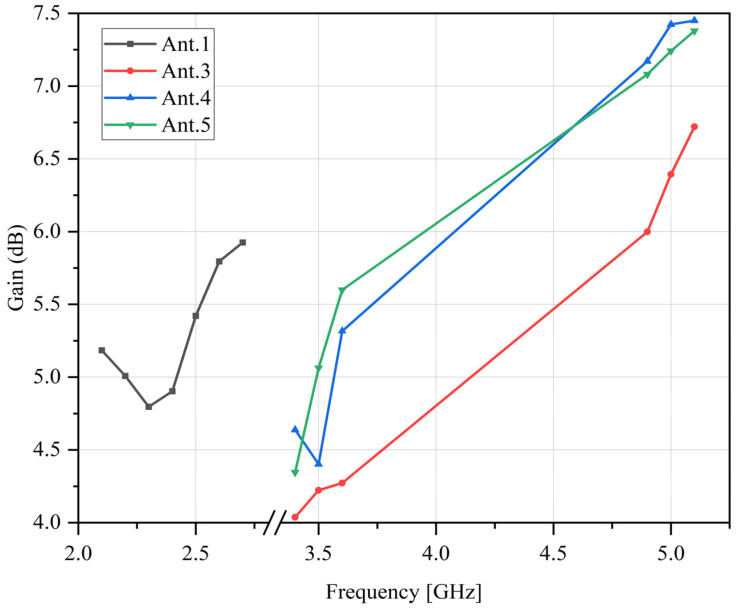
Gain of the antenna.

Since both sides of the antenna are symmetrical, the radiation direction of one side of the antenna is shown in Figure 14. At 2500 MHz, the decoupling structure added makes antenna 1 have lateral radiation characteristics, so the isolation of antenna 1 and antenna 2 is good. At 3500 MHz, antenna 4 has omnidirectional radiation characteristics. The gain of the antenna is shown in Figure 15. It can be observed that the gain of the antenna is more than 3 dB in the operating band, and the actual gain of the antenna will be reduced because of the simulation result.

## 4. Prototype and Comparison

**Figure 16:**
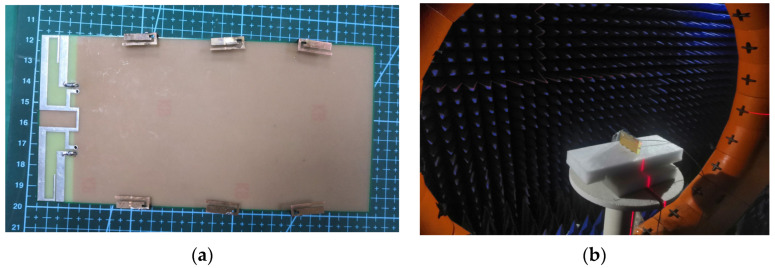
Photographs of the fabricated prototype, (**a**) fabricated prototype, and (**b**) verification environment.

The proposed antenna has also been fabricated and verified in the laboratory. Figure 16 shows photographs of the fabricated antenna and its verification environment.

**Figure 17:**
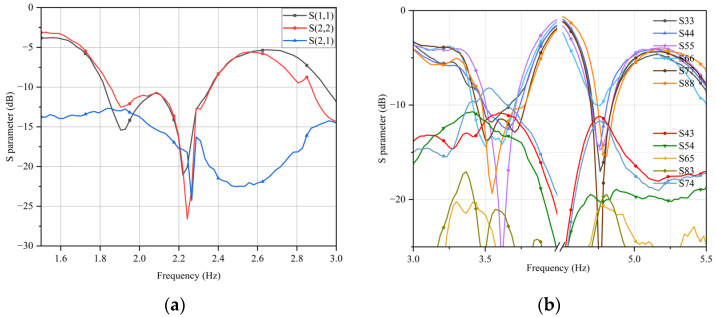
Scattering parameters of prototype, (**a**) 4G antenna prototype, and (**b**) 5G antenna prototype.

After completing the calibration, the scattering parameters of the antenna were measured in sequence, as shown in Figure 17. Due to the symmetry of the antenna on both sides, the return loss of all antennas and the coupling between some antennas were given. The test results show that the operating frequency band of antennas are 2000–2600 MHz, 3400–3600 MHz, and 4800–5000 MHz. The return loss of the antennas within the operating frequency and the isolation between adjacent units on the same side or symmetric adjacent units on both sides’ bands are greater than 10 dB. Except for a small amount of frequency deviation, the test data of the scattering parameters fits well with the simulated data and meets the working requirements. The analysis shows that the slight frequency deviation of the two units is caused by the noise in the test environment or poor welding accuracy.

**Figure 18:**
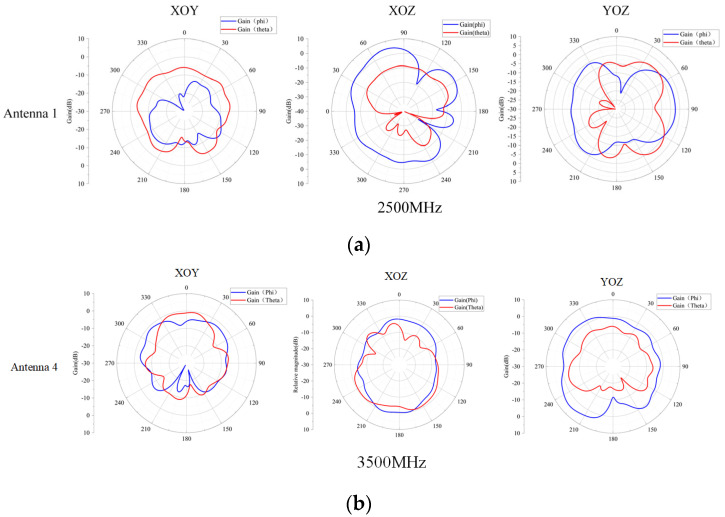
(**a**) Radiation pattern of the prototype antenna 1. (**b**) Radiation pattern of the prototype antenna 4.

Due to the symmetry of the antenna on both sides, only one side of the antenna was tested, which includes the radiation patterns and gain of antennas 1 and 4. The radiation patterns of the antennas were measured and shown in Figure 18.

**Figure 19:**
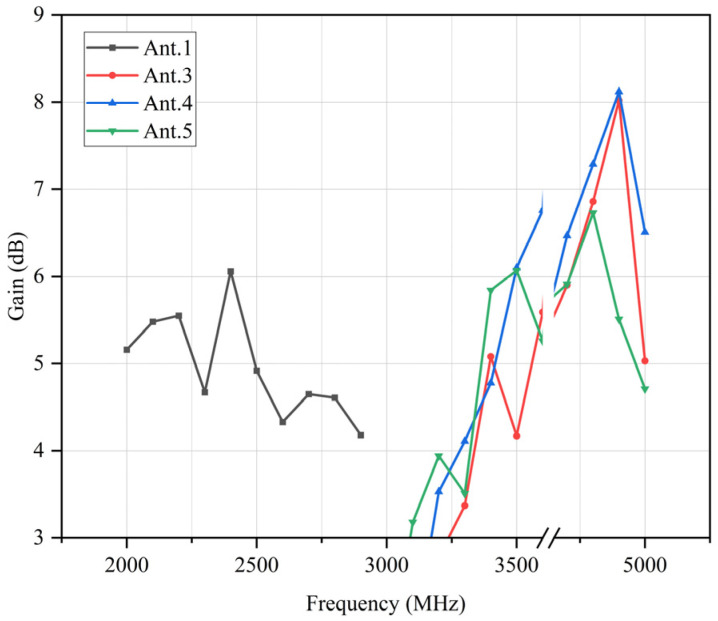
Gain of the prototype antenna.

The gain of the antenna is measured and shown in Figure 19. In the operating frequency band of antennas 1 and 3–5, which are 2000–2600 MHz, 3400–3500 MHz, and 4800–5000 MHz, the gain of the antennas is greater than 3 dB which meets the design requirements.

The performance comparison of the proposed eight-element mobile MIMO antenna with other mobile MIMO antennas is presented in Table 1.

The proposed antenna covers 2.0–2.6, 3.4–3.6, and 4.8–5.0 GHz, which is much wider than the antenna in Table 1. Meanwhile, miniaturization of the proposed antenna has also been considered; the size of 70 × 140 × 0.8 mm^3^ suits most of the smartphones. The standard parameters such as ECC and isolation still maintain an excellent level despite the burden of the wide band. The wide band also produces more gain with 5–6 dB, 3.5–6 dB, and 7–8 dB.

## 5. Conclusions

This paper proposes an eight-element multimode mobile phone antenna consisting of two 4G antenna elements and six 5G antenna elements. Within the operating bandwidth of the antenna, the antenna elements are well matched, and the return loss of the antenna is greater than 10dB. The coupling between antenna elements is small, and the isolation between any two antenna elements is larger than 10 dB. By calculation, the envelope correlation coefficient between any two antenna elements is less than 0.1, indicating that the MIMO antenna has good diversity characteristics and small diversity gain. The simulation proves that the MIMO antenna is less affected by the human body when approaching the head and hand, and the radiation performance remains basically unchanged. The 4G antenna covers 4G mobile communication frequency bands such as B1, B38, B40, B41, and n41 frequency bands of 5G. The 5G antenna can work on the two frequencies of 3400–3600 MHz and 4800–5000 MHz, which can cover the n78 and n79 bands of 5G mobile communications, respectively, and are the two most mature frequency bands for sub-6 GHz applications.

## Figures and Tables

**Table 1:** Comparison of the proposed antennas with other works for 5G smartphones.

Reference	Year	Frequency (GHz)	Size (mm^3^)	ECC	No. of Ports	Isolation (dB)	Gain (dB)
[14]	2021	2.37–5.85	70 × 145 × 0.2	<0.05	4	17.5	4–5.5
[15]	2021	2.3–6.5	75 × 150 × 0.8	0.01	6	>20	>3
[16]	2022	3.18–4.4	4 × 38 mm^2^	<0.17	8	>12.25	>2.81
[17]	2023	3.3–5.0	7 × 30 × 2	<0.08	8	>10	–
[18]	2022	3.3–7.1	22 × 22 mm^2^	<0.07	8	>12	4.6–6.2
Proposed	2023	2.0–2.6/3.4–3.6/4.8–5.0	70 × 140 × 0.8	<0.1	8	>10	5–6/3.5–6/7–8

## Data Availability

Data available on request due to restrictions eg privacy or ethical. The data presented in this study are available on request from the corresponding author. The data are not publicly available due to privacy or ethical.

## References

[1] Ikram M., Al Abbas E., Nghia N.T., Sayidmarie K.H., Abbosh A. (2019). Integrated Frequency-Reconfigurable Slot Antenna and Connected Slot Antenna Array for 4G and 5G Mobile Handsets. IEEE Trans. Antennas Propag..

[2] Giambene G., Kota S., Pillai P. (2018). Satellite-5G Integration: A Network Perspective. IEEE Netw..

[3] Al-Dulaimi A., Al-Rubaye S., Ni Q., Sousa E. (2015). 5G Communications Race. IEEE Veh. Technol. Mag..

[4] Behrad S., Bertin E., Crespi N. (2019). A survey on authentication and access control for mobile networks: From 4G to 5G. Ann. Telecommun..

[5] Wang Y., Xu J., Jiang L.S. (2014). Challenges of System-Level Simulations and Performance Evaluation for 5G Wireless Networks. IEEE Access.

[6] Cui L., Guo J.L., Liu Y., Sim C.Y.D. (2019). An 8-Element Dual-Band MIMO Antenna with Decoupling Stub for 5G Smartphone Applications. IEEE Antennas Wirel. Propag. Lett..

[7] Liu Y., Ren A.D., Liu H., Wang H.Y., Sim C.Y.D. (2019). Eight-Port MIMO Array Using Characteristic Mode Theory for 5G Smartphone Applications. IEEE Access.

[8] Liu D.Q., Zhang M., Luo H.J., Wen H.L., Wang J. (2018). Dual-Band Platform-Free PIFA for 5G MIMO Application of Mobile Devices. IEEE Trans. Antennas Propag..

[9] Chen Q.G., Lin H.W., Wang J.P., Ge L., Li Y.J., Pei T.Q., Sim C.Y.D. (2019). Single Ring Slot-Based Antennas for Metal-Rimmed 4G/5G Smartphones. IEEE Trans. Antennas Propag..

[10] Ikram M., Nguyen-Trong N., Abbosh A. (2020). Hybrid Antenna Using Open-Ended Slot for Integrated 4G/5G Mobile Application. IEEE Antennas Wirel. Propag. Lett..

[11] Ban Y.L., Li C., Sim C.Y.D., Wu G., Wong K.L. (2016). 4G/5G Multiple Antennas for Future Multi-Mode Smartphone Applications. IEEE Access.

[12] Wong K.L., Kang T.W., Tu M.F. (2011). Internal mobile phone antenna array for LTE/WWAN and LTE MIMO operations. Microw. Opt. Technol. Lett..

[13] Ban Y.L., Chen Z.X., Chen Z., Kang K., Li J.L.W. (2014). Decoupled Closely Spaced Heptaband Antenna Array for WWAN/LTE Smartphone Applications. IEEE Antennas Wirel. Propag. Lett..

[14] Kulkarni J., Alharbi A.G., Desai A., Sim C.Y.D., Poddar A. (2021). Design and Analysis of Wideband Flexible Self-Isolating MIMO Antennas for Sub-6 GHz 5G and WLAN Smartphone Terminals. Electronics.

[15] Kulkarni J., Dhabre S., Kulkarni S., Sim C., Cengiz K. Six-Port Symmetrical CPW-Fed MIMO Antenna for Futuristic Smartphone Devices. Proceedings of the 2021 6th International Conference for Convergence in Technology (I2CT).

[16] Zhou H., Wu D., Zhu M.M., Qiu Y., Yu G.L., Zhou H.M. (2022). Wideband Low-Profile 8 × 8 MIMO Antenna Based IFA Pair for Ultrathin 5G Smartphones. Int. J. Antennas Propag..

[17] Qian B.Y., Huang X.Y., Chen X.M., Chang L., Wei K.P., Al-Hadi A.A., Kishk A.A. (2023). Wideband self-decoupled MIMO antenna element based on coupled-loop antenna for 5G smartphones. J. Electromagn. Waves Appl..

[18] Sghaier N., Latrach L. (2022). Design and analysis of wideband MIMO antenna arrays for 5G smartphone application. Int. J. Microw. Wirel. Technol..

[19] Karaboikis M.P., Papamichael V.C., Tsachtsiris G.F., Soras C.F., Makios V.T. (2008). Integrating compact printed antennas onto small diversity/MIMO terminals. IEEE Trans. Antennas Propag..

